# What Blood Pressure Does Not Reveal: Intimate Partner Violence Among Adults with Hypertension

**DOI:** 10.3390/healthcare14203496

**Published:** 2026-10-17

**Authors:** Abrar I. Aljohani, Turki S. Alqurashi, Samar M. Alotaibi

**Affiliations:** 1 Department of Clinical Laboratory Sciences, College of Applied Medical Sciences, Taif University, Taif 21944, Saudi Arabia; 2 Department of Social Work, Al-Lith University College, Umm Al-Qura University, Makkah 28429, Saudi Arabia; tsmqurashi@uqu.edu.sa; 3 Maternal and Child Health Nursing Department, College of Nursing, Shaqra University, Al Dawadmi11911, Saudi Arabia; samar_m@su.edu.sa

**Keywords:** intimate partner violence, hypertension, IPV, cardiovascular health

## Abstract

Background: Few studies have characterized the patterns and sociodemographic correlates of reported intimate partner violence (IPV) behaviors among people with hypertension. This study characterized IPV among individuals with physician-diagnosed hypertension in Saudi Arabia. Methods: This cross-sectional study recruited 471 individuals with hypertension from multiple healthcare facilities in Saudi Arabia using convenience sampling. IPV was assessed using questions covering physical, emotional/verbal, sexual, and controlling behaviors. “Any reported IPV behavior” was defined as at least one behavior in any domain; domain-specific and multidomain IPV were also examined. The IPV items were not uniformly anchored to a standardized 12-month or lifetime reference period. Sociodemographic differences were assessed using Pearson’s chi-square tests. As complete separation occurred in some categories, Firth’s bias-reduced penalized logistic regression was used for exploratory estimation of crude odds ratios (ORs) and adjusted ORs (aORs) with 95% confidence intervals (CIs). Results: Overall, 278 (59.0%) participants reported at least one assessed IPV behavior. Controlling behavior was reported by 212 participants (45.0%), followed by emotional/verbal (28.2%), physical (26.8%), and sexual IPV (13.0%). Multidomain IPV was reported by 33.8%. IPV was reported by 75.5% of women and 33.5% of men. Men had lower odds of reporting IPV than women (aOR = 0.09, 95% CI: 0.04–0.18). Age-related estimates were unstable because all participants aged 36–49 years reported IPV, resulting in complete separation. Conclusions: Reported IPV behaviors were common in this Saudi sample. Controlling behavior was the most frequently reported domain, and about one-third reported multidomain IPV, indicating the importance of recognizing non-physical forms of IPV among people with hypertension. Larger, representative studies using standardized IPV measures are needed to confirm these findings and to examine potential relationships between IPV and cardiovascular health outcomes.

## 1. Introduction

Intimate partner violence (IPV) is a significant public health and human rights issue that encompasses controlling behaviors, psychological or emotional abuse, sexual coercion, and physical aggression in a current or former intimate relationship [1]. Violence perpetrated by intimate partners is among the most prevalent forms of violence against women worldwide. The World Health Organization (WHO) estimates that approximately one in three women will experience physical and/or sexual violence by an intimate partner, or sexual violence by a non-partner, during their lifetime. The prevalence of such violence varies considerably across sociocultural and geographic contexts. This is particularly relevant regionally, where lifetime physical and/or sexual IPV is estimated to affect approximately 29% of ever-partnered women in Western Asia [2].

The implications of IPV extend beyond acute physical harm and include adverse mental, reproductive, behavioral, and chronic physical health outcomes. Recurrent exposure to interpersonal violence is a potential major source of sustained psychosocial stress, which may particularly affect cardiovascular health [3]. Chronic activation and dysregulation of neuroendocrine and autonomic stress responses, inflammatory processes, and adverse health behaviors have been proposed as pathways linking violence exposure to cardiovascular risk [4]. Consequently, there has been a growing focus on the potential association between violence exposure and hypertension and other cardiovascular outcomes [5].

However, the association between IPV and hypertension is not well understood. A 2025 systematic review and meta-analysis of 11 studies including over 153,000 participants found an overall association between violence exposure and hypertension, with significant associations for physical and sexual violence, although the pooled association for IPV did not reach statistical significance [5]. More recently, a 2026 systematic review and meta-analysis focusing on IPV against women and hypertension reported a modest association between lifetime exposure to any IPV and hypertension. The included studies differed in demographic characteristics, research design, domains of IPV measured, and exposure durations (including previous 12-month and lifetime measurements), which limited comparisons between studies [6]. In the current investigation, some of this heterogeneity was reduced by using the same behavior-based IPV evaluation and domain criteria for all participants within a single clinical group of individuals with physician-diagnosed hypertension. Together, these findings suggest that the correlation between hypertension and IPV is still subject to uncertainty, while also pointing to critical gap in understanding the burden and patterns of IPV in individuals who have already developed hypertension.

IPV is an important public health concern in Saudi Arabia; however, it has been incompletely characterized. Earlier Saudi studies have reported widely varying prevalence estimates, reflecting heterogeneity in demographics, settings, definitions, instruments, and exposure durations. For example, a study of 497 women attending basic healthcare centers in Taif reported a past-year IPV prevalence of 11.9%, whereas a survey of 720 ever-married women attending primary care in Riyadh reported a 20% past-year prevalence of domestic violence [7,8]. More recently, a study of 403 Saudi women attending primary healthcare centers in the Aljouf region reported that 30.3% of participants experienced IPV [9]. A systematic review of Saudi research reached similar conclusions, noting that existing prevalence estimates were unreliable, that definitions and measurement methods varied greatly, and that nationally representative evidence was lacking [10]. A second scoping review found considerable variability in sampling techniques, measurement tools, study populations, and reported frequencies across Saudi studies [11].

Most Saudi literature has focused on women recruited from general primary healthcare or community settings [7], with limited evidence specifically characterizing adults with established hypertension. Consequently, the burden and patterns of IPV within this clinical population remain poorly described. This is a clinically important gap, since people with hypertension see healthcare providers regularly, which allows opportunities to detect psychological vulnerabilities that might otherwise remain unrecognized. Moreover, studying IPV in a hypertensive population offers a perspective distinct from research focused solely on whether IPV is associated with an increased risk of incident hypertension. Rather than treating hypertension as an endpoint, this approach describes the burden of IPV in an established hypertensive population and identifies the sociodemographic categories in which IPV is most often reported.

Another limitation of much of the available data is the tendency to report either an overall measure of IPV or a limited number of violence categories. However, IPV is multifaceted, and multiple forms of abuse may coexist in the same relationship. Characterizing individual behaviors, prevalence, and exposure across specific IPV domains may provide a more meaningful picture of its impact than a single binary prevalence estimate. This is especially true in contexts where controlling, emotional, sexual, or other non-physical forms of IPV are less likely to be identified or reported. The current evidence base is limited by variation in the definition and assessment of IPV, as noted in the Saudi studies [10,11].

Accordingly, this study aimed to characterize the patterns of reported IPV behaviors among adults with physician-diagnosed hypertension in Saudi Arabia. Specifically, we aimed to (1) estimate the proportion of participant reporting at least one assessed IPV behavior, (2) describe the proportions reporting physical, emotional/verbal, sexual, and controlling-behavior domains, (3) characterize the co-occurrence of reporting IPV across multiple domains, and (4) examine sociodemographic factors associated reporting any IPV behavior. The present study seeks to contribute to the literature on IPV and cardiovascular health by characterizing IPV among individuals with established hypertension at the behavioral, domain, and overall IPV levels. Because the study included only individuals with physician-diagnosed hypertension and no normotensive control group, it aimed to characterize reported IPV within this population rather than to investigate whether IPV is associated with the incidence or development of hypertension.

## 2. Materials and Methods

### 2.1. Study Design and Participants

A cross-sectional study was conducted among individuals with physician-diagnosed hypertension living in Saudi Arabia. Eligible participants were ≥18 years of age, had a prior physician diagnosis of hypertension, and were able to complete the study questionnaire. Individuals who reported being single at the time of recruitment were excluded based on the initial study eligibility criteria, as the IPV evaluation was designed to analyze behaviors in current or prior marital partnerships. In the questionnaire, the term “partner” referred to the current spouse for married participants and the former spouse for divorced individuals. The study did not determine if individuals who were single at recruitment had previously been in dating or other non-marital intimate relationships. As a result, the analytic sample included participants who were married or divorced at the time of study. Additionally, other exclusion criteria included having no confirmed diagnosis of hypertension, reporting only pre-hypertension or elevated blood pressure without a physician diagnosis, and failing to complete the required information for the study analyses or to provide informed consent.

Data were collected using questions adapted from the Survey Module on Violence against Women developed for the United Nations Economic Commission for Europe [12]. The original survey questions may not be suitable for research in Saudi Arabia given the country’s cultural context, so applying the internationally recognized IPV survey module directly to the Saudi setting would be inappropriate [11]. Therefore, the questionnaire was adapted for Saudi men and women [12,13]. This was intended to minimize ascertainment bias, characterize the proportions and patterns of reported IPV behaviors across different domains, and ensure reliable data collection.

A non-probability convenience sampling method was used. The sample size was estimated using the Raosoft sample size calculator with a 50% response distribution, 5% margin of error, and 95% confidence level. We assumed a response distribution of 50% as a conservative choice, since it yields the maximum required sample size when the expected proportion is unknown. The minimum required sample size was 377 individuals. This sample-size calculation was intended for descriptive estimation of the proportion reporting IPV behaviors and not for statistical power or sample adequacy for the multivariable regression analyses.

### 2.2. Recruitment and Data Collection

Data were collected using an anonymous, self-administered online questionnaire in Arabic via Google Forms. Recruitment was mostly from eight outpatient cardiology clinics and primary healthcare centers in various locations across Saudi Arabia. These facilities routinely examine and manage cardiovascular diseases, including hypertension. Nurses and reception staff at these settings identified potential participants and provided information about the study, along with a QR code or electronic link to the questionnaire, as part of usual clinical treatment.

Five charitable cardiac organizations were also included to supplement recruitment to widen the study’s reach. These organizations were requested to send the invitation only to people with a physician-confirmed diagnosis of hypertension, in keeping with the eligibility criteria used at the participating healthcare institutions. Screening questions at the beginning of the questionnaire also confirmed eligibility.

Participation was voluntary, and the questionnaire was completed without the involvement of healthcare professionals. All study variables were mandatory fields in the online questionnaire, so all submitted questionnaires contained complete data for the variables used in the analysis. However, because requiring responses to all items may have discouraged completion among people who are unwilling or unable to answer certain questions, it could constitute a source of non-response bias.

**Figure 1:**
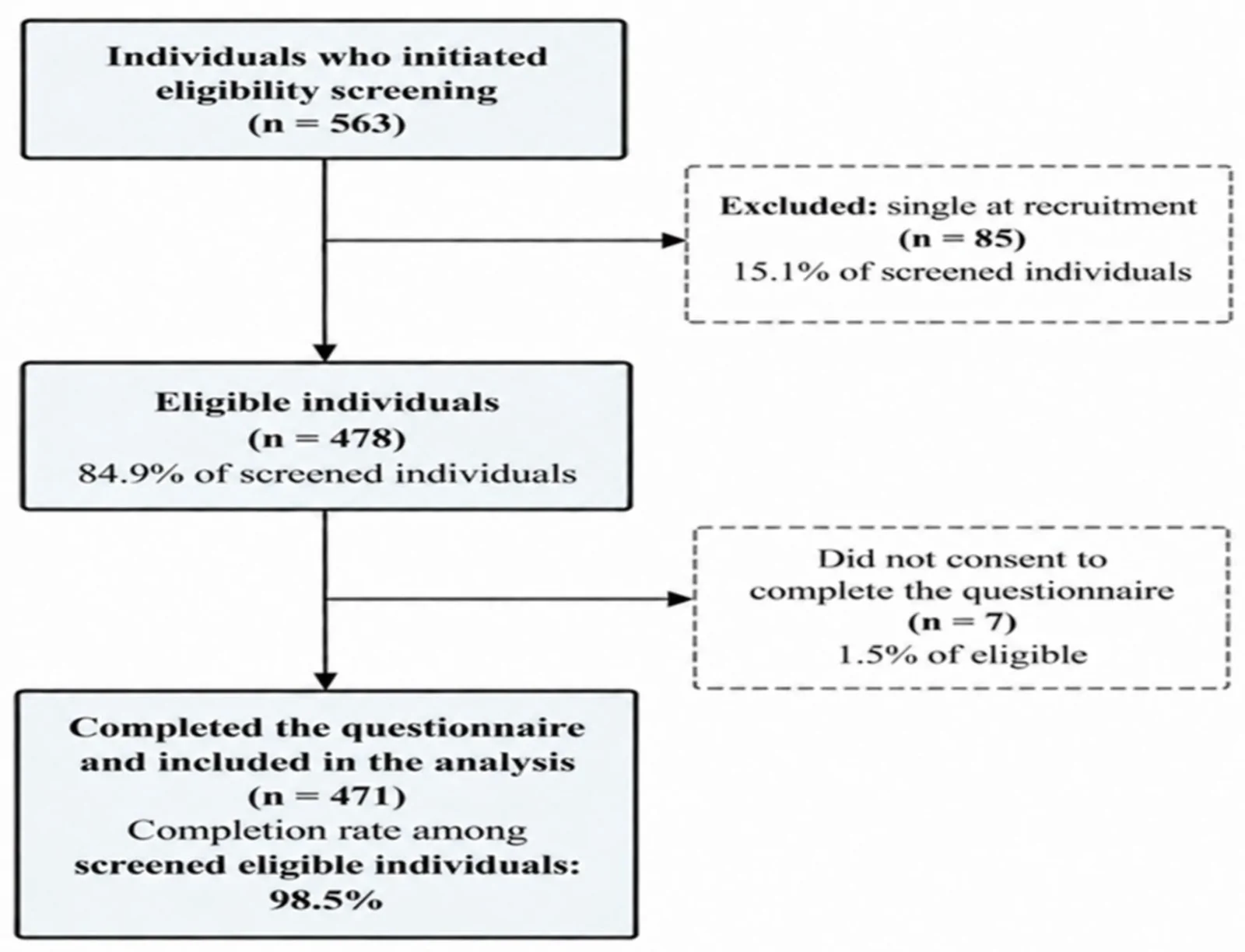
Flow diagram of participant recruitment and inclusion in the study.

In total, 563 individuals-initiated eligibility screening. These were reduced to 478 eligible individuals, as 85 were deemed ineligible. Seven eligible individuals declined to provide consent; however, 471 completed the questionnaire and were included in the analysis, resulting in a completion rate of 98.5% among screened eligible individuals (471/478) (Figure 1). The overall response rate among all individuals exposed to the invitation could not be calculated, as individuals who received or viewed the invitation but did not initiate eligibility screening could not be enumerated.

### 2.3. Questionnaire Adaptation, Validity and Reliability

The IPV evaluation was based on the United Nations Economic Commission for Europe (UNECE) Survey Module on Violence against Women. The original module was designed for women and had gender-specific language, detailed instructions for the interviewer, many follow-up questions, and context-dependent items; hence, it was not used verbatim. Instead, the instrument was converted into a short self-administered Arabic questionnaire that would be appropriate for both men and women respondents in the Saudi setting. The Arabic survey utilized the phrase “partner,” which was interpreted as husband or wife, thereby avoiding the gendered language of the original module.

The adaptation aimed to maintain the behavior-based nature of the UNECE instrument while prioritizing items that were considered explicit, relevant, and appropriate for gender-neutral administration. The major modifications were aimed at promoting gender-neutral self-administration, reducing respondent burden, and improving cultural applicability while maintaining the behavior-based structure of the original module. The final IPV assessment consisted of 15 items that addressed four domains: controlling, emotional/verbal, sexual, and physical behaviors. Four of the six UNECE physical-violence behaviors were retained: slapping or throwing a potentially harmful item; pushing, shoving, or pulling hair; kicking or dragging; and choking or intentionally burning. The shortened adapted version did not include the UNECE items that pertain to the use of a weapon, threatening, or striking with a fist or another object. The behaviors in the original UNECE module are closely correlated with the physical items that were retained.

Three of the four UNECE emotional/verbal items were maintained with gender-neutral language, which includes deliberate intimidation, belittling or humiliating the participant in front of others, and insulting or making the participant feel bad. The original UNECE item, which addressed verbal threats to physically harm the respondent or another person, had been modified during cultural adaptation to evaluate behaviors that suggest relationship instability, such as threats or indications of separation, divorce, or abandonment. As a result, this item was classified as an adaptation rather than a direct translation. This relationship-instability item is explicitly included in the Arabic questionnaire.

All three UNECE sexual-violence behaviors were retained because they could be directly translated into gender-neutral, behavior-based language without significantly altering the underlying construct: forced sexual intercourse, unwanted intercourse due to fear of the partner, and being forced to perform an unwanted or humiliating sexual act.

The controlling-behavior domain underwent a more significant degree of adaptation due to the fact that these behaviors are particularly sensitive to social and cultural context. The UNECE module itself acknowledges that controlling and emotional-behavior items are still undergoing methodological development and that economic violence necessitates context-specific adaptation. Consequently, the two UNECE items that pertain to the restriction of contact with friends and family were merged into a single item, while several items that pertain to monitoring, jealousy, suspicion, and permission to seek healthcare were omitted. The adapted survey instead encompassed behaviors that were deemed pertinent to autonomy and partner control in the intended study context, such as the restriction of employment or study, the restriction of leaving the home, and the control or taking of money without consent. The Arabic questionnaire shows these four items regarding controlling behavior.

The UNECE module also includes item-specific requests that relate to the frequency and recurrent occurrence of each behavior, as well as whether it occurred within the past 12 months. The present questionnaire was primarily designed to identify whether each assessed IPV behavior was reported. and to characterize overall and domain-specific IPV patterns, rather than to quantify frequency or assess IPV within a standardized 12-month reference period. Consequently, these detailed follow-up questions were not retained. These modifications could have altered the classification of reported IPV. Because a participant was classed as reporting a domain if at least one assessed behavior within that domain was endorsed, omitting an original UNECE item may result in non-classification of a participant whose only IPV experience comprised the omitted behavior. In contrast, updated or context-specific elements may record experiences that differ from the original UNECE module. Merging items may also limit the ability to distinguish between various forms of controlling behavior. As a result, the classifications in the current study reflect the adapted 15-item assessment and should not be considered directly equivalent to classifications generated from the full UNECE instrument. Formal cognitive interviewing was not conducted. The adapted gender-neutral version had not previously undergone comprehensive psychometric validation specifically among Saudi men and women.

The questionnaire was translated into Arabic and back-translated into English by independent translators to ensure conceptual and linguistic equivalence. Content validity and cultural appropriateness were assessed by two experts with relevant experience in family medicine and IPV research. The questionnaire was pilot-tested among 32 participants to evaluate clarity, comprehension, and practicality. Internal consistency was assessed using Cronbach’s alpha in the study sample. Internal consistency for the entire 15-item IPV scale was excellent (Cronbach’s α = 0.906). Domain-specific reliability was acceptable to good (α = 0.827 for physical IPV, 0.846 for emotional/verbal IPV, 0.897 for sexual IPV, and 0.700 for controlling behaviors). The coefficients were interpreted exclusively as indicators of internal consistency. The overall alpha was not interpreted as evidence of the instrument’s construct validity or unidimensionality, or comprehensive psychometric validation, as the 15 items represent conceptually distinct IPV domains.

### 2.4. Sociodemographic Characteristics

At the participant level, sociodemographic variables included gender, age group, marital status, educational level, and occupational status. Gender was coded as women or men. Age was categorized as 18–25, 26–35, 36–49, and ≥50 years. Marital status was classified as married or divorced. Educational level was categorized as less than secondary education, secondary education, bachelor’s degree, or master’s degree. Occupational status was classified as housewife/unemployed, employed, student, or retired.

Information was also collected on participants’ partners. Partner educational level was classified as less than secondary education, secondary education, bachelor’s degree, or master’s degree, and partner occupational status as housewife/unemployed, employed, or student. These factors were included to assess the sociodemographic distribution of reported IPV behaviors and possible variations in the proportion reporting IPV by participant and partner characteristics.

### 2.5. Assessment of IPV

IPV was assessed using questions covering four domains: physical IPV, emotional/verbal IPV, sexual IPV, and controlling behavior. The term “partner” was used to refer to the current spouse for married participants, while it was used to refer to the former spouse for divorced participants. To promote clarity and encourage disclosure, participants were given culturally relevant, non-stigmatizing explanations of the behaviors under examination before they completed the IPV section of the questionnaire.

Physical IPV was assessed using questions about experiences of being slapped or having an object thrown that could cause harm; being pushed, shoved, or having one’s hair pulled; being kicked or dragged; and being deliberately choked or burned.

Emotional/verbal IPV was assessed using items on being insulted or made to feel bad about oneself, being belittled or humiliated in front of others, being deliberately frightened or intimidated, and being threatened with relationship instability or separation.

Sexual IPV was assessed using items on being forced to have sexual intercourse while unwilling; having sexual intercourse out of fear of the partner’s reaction; and being compelled to perform an undesirable, degrading, or humiliating sexual act.

Controlling behavior included restricting contact with friends or family, preventing the participant from leaving the house, obstructing employment or education, and controlling or seizing money without permission. Responses to each IPV item were coded as 0 = no and 1 = yes. This study used individual behavior-based responses to define reported IPV experiences.

The behavior-based IPV questions were not consistently linked to a standardized exposure period, such as the previous 12 months or a clearly defined lifetime interval. As a result, the current study reflects whether participants reported experiencing each assessed IPV behavior rather than estimating IPV within a standardized reference period. Throughout the manuscript, the term “reported IPV behaviors” is used to describe these results.

### 2.6. Definition of IPV Outcomes

A binary variable was created for each of the four IPV domains. A participant was classified as positive for a domain if they reported at least one behavior within that domain and negative if none of the behaviors within the domain were reported.

The primary outcome—any reported IPV behavior—was defined as reporting at least one examined behavior in any of the four IPV domains. Participants reporting no behavior across all four domains were considered not to have reported any assessed IPV behavior.

The number of IPV domains reported by each participant (range 0–4) was also calculated. To capture the degree of overlap between the different types of IPV, participants were categorized into three mutually exclusive groups: no IPV, single-domain IPV, and multidomain IPV.

The multidomain measure indicates the breadth of IPV experiences across domains and should not be interpreted as a direct measure of frequency, chronicity, or within-domain severity, none of which were assessed in the current study.

### 2.7. Statistical Analysis

Descriptive statistics were used to summarize the sociodemographic characteristics of participants and the frequency and patterns of reported IPV behaviors. Categorical variables were reported as frequencies and percentages. Any reported IPV behavior was defined as reporting at least one behavior in the physical, emotional/verbal, sexual, or controlling-behavior domains. The frequency of individual IPV behaviors and IPV domains was estimated as the percentage of participants reporting the corresponding behavior or domain. For the principal descriptive proportions, 95% confidence intervals were calculated using the Wilson score method.

Pearson’s chi-square test was used to assess bivariable correlations between sociodemographic factors and reporting any IPV behavior. The assumptions of the chi-square test were checked by examining expected cell frequencies, and exact testing was used when these assumptions were not met. Sociodemographic comparisons were computed as row percentages within each group. All tests were two-sided, and a *p* value < 0.05 was considered statistically significant.

Conventional maximum-likelihood logistic regression was deemed unsuitable for the primary multivariable analysis because of the complete separation observed in the data, particularly in the 36–49-year age group, where all participants reported at least one IPV behavior. Therefore, Firth’s bias-reduced penalized logistic regression was used to estimate associations between sociodemographic factors and reporting any IPV behavior. Crude odds ratios (ORs) were calculated using separate univariable Firth logistic regression models.

Participant-level sociodemographic factors evaluated in the multivariable analysis included gender, age group, marital status, educational level, and occupational status. Categorical predictors were coded as indicator variables, with women, participants aged 18–25 years, married participants, those with less than secondary education, and housewives/unemployed participants as the respective reference groups.

Candidate sociodemographic covariates were chosen based on their conceptual relevance to IPV and frequent examination as potential correlates in previous IPV research, rather than purely on bivariable statistical significance. Gender, age, marital status, educational level, and employment status were therefore investigated as possible variables prior to multivariable model design. Variance inflation factors (VIFs) were used to examine multicollinearity among potential predictors before finalizing the multivariable model. When occupational status was included alongside the other participant-level variables, there was evidence of considerable multicollinearity, with VIFs ranging from 1.87 to 12.51 and the largest values among the age-group indicators. With occupational status removed, VIFs ranged from 1.60 to 3.69. Therefore, the main multivariable Firth model included gender, age group, marital status, and educational level. The variable “occupational status” was excluded from the main model because its inclusion caused multicollinearity and instability of the adjusted estimates. The more parsimonious model was retained as it maintained the main sociodemographic covariates.

As a sensitivity analysis, occupational status was added to the main model to test the robustness of the adjusted associations to its inclusion. The adjusted estimates were examined for changes in magnitude, direction, and precision, with particular attention to estimates affected by sparse covariate patterns and separation. Results of the regression analyses were reported as crude ORs and adjusted ORs (aORs) with 95% confidence intervals (CIs) and two-sided *p*-values. The multivariable regression analyses were considered exploratory and adjusted values were interpreted with caution given sparse outcome patterns, complete separation and sensitivity of coefficients to model specification.

Internal consistency of the IPV assessment was evaluated using Cronbach’s alpha for each IPV domain. The individual IPV items were coded dichotomously (0 = no, 1 = yes); the resulting alpha coefficients are, therefore, equivalent to Kuder–Richardson Formula 20 coefficients for dichotomous items.

Statistical analysis was performed using IBM SPSS Statistics (version 31.0.2). Firth penalized logistic regression was performed using the R package logistf through the SPSS extension.

### 2.8. Ethical Considerations

This study was part of a larger study examining the association of non-communicable diseases with family violence, including IPV and child maltreatment. The study received ethics approval from the Biomedical Research Ethics Committee of Umm Al-Qura University (approval no. HAPO-02-K-012-2025-03-2622).

The electronic study invitation explained the study’s aim and procedures to potential participants. Participation was entirely voluntary, and electronic informed consent was obtained before participants were given access to the questionnaire. Because the IPV-related questions were sensitive, a notice was included on the invitation and consent page to advise participants that the questionnaire contained sensitive information. Participants who felt distressed or uncomfortable while completing the questionnaire were invited to seek help from local counseling or family-service organizations. The questionnaire was self-administered online; however, since the completion was independent, the study team was unable to directly verify the privacy of the participants’ physical surroundings. Furthermore, since no personally identifying information was collected and replies were anonymous, the study team was unable to identify individuals who reported continuing violence and so was unable to conduct a specific individualized safety or referral response. No personally identifiable information was sought. Responses were anonymized before analysis, and access to the study data was restricted to the research team. Electronic data was stored in password-protected files accessible only to the investigators.

## 3. Results

### 3.1. Study Participant Characteristics

The study included 471 participants. Most were women (60.7%), aged ≥50 years (50.1%), and married (77.7%). Among the 65 participants aged 18–25 years, 38 (58.5%) were married and 27 (41.5%) were divorced. These proportions reflect the marital-status distribution within the present convenience sample and should not be interpreted as population-level estimates for young adults in Saudi Arabia. The educational level and occupational status of participants and their partners are presented in Table 1.

### 3.2. Patterns of Reported Individual IPV Behaviors

Table 2 presents the individual IPV behaviors across domains. The most frequently reported behaviors were in the emotional/verbal and controlling domains, notably being insulted or made to feel bad about oneself (26.3%), restriction of contact with friends or family (24.2%), and being prevented from employment or study (23.8%). The most frequently reported physical behavior was being slapped or having something thrown that could hurt (23.8%). Sexual IPV behaviors were less frequently reported, ranging from 6.4% to 12.5%.

### 3.3. Overall and Domain-Specific Reporting of IPV Behaviors

In all, 59.0% of participants reported at least one of the assessed IPV behaviors (Table 3). Controlling behavior was the most frequently reported IPV domain (45.0%), followed by emotional/verbal (28.2%) and physical IPV (26.8%), while sexual IPV was the least common (13.0%). Multidomain IPV was reported by 33.8% of participants.

### 3.4. Sociodemographic Factors Associated with Reporting Any IPV Behavior

Bivariable analyses indicated significant associations between reporting any IPV behavior and gender, age group, marital status, educational level, occupational status, partner’s educational level, and partner’s occupational status (all *p* < 0.001; Table 4). IPV was reported more often by women (75.5%), participants aged 36–49 years (100.0%), those with secondary education (82.4%), and housewives/unemployed participants (80.5%). Notably, all participants aged 36–49 years (91/91) and all those whose partners had a bachelor’s degree (27/27) reported at least one IPV behavior.

### 3.5. Multicollinearity Assessment

The first candidate multivariable model included gender, age group, marital status, educational level, and occupational status. Multicollinearity diagnostics indicated high VIFs, particularly for age and occupational status. The VIFs ranged from 1.87 to 12.51, with the highest value in the ≥50 years age group (VIF = 12.51). The VIFs for the 26–35- and 36–49-year age groups were 7.22 and 8.06, respectively, and those for the occupational status indicators ranged from 4.83 to 5.94.

After occupational status was removed, the VIFs decreased substantially, with VIFs ranging from 1.60 to 3.69 compared with 1.87 to 12.51 in the initial model. The VIF was 1.80 for gender, 2.05–3.69 for age categories, 1.60 for marital status, and 1.68–2.60 for educational categories. Because multicollinearity was reduced, the model including gender, age group, marital status, and educational level was retained as the main adjusted model. In a sensitivity analysis, occupational status was then included to assess the robustness of the primary-model findings.

### 3.6. Firth Logistic Regression

Crude and adjusted associations between participant sociodemographic variables and reporting any IPV behavior are presented in Table 5. We used Firth bias-reduced penalized logistic regression because we detected complete separation in the 36–49-year age group, where all 91 participants reported at least one IPV behavior.

In the main adjusted model, reporting any IPV behavior was associated with gender, marital status, and educational level; however, the age-group estimates revealed significant instability owing to complete separation. Compared with women, men had significantly lower adjusted odds of reporting any IPV behavior (aOR = 0.09, 95% CI: 0.04–0.18; *p* < 0.001). Divorced participants also had lower adjusted odds than married participants (aOR = 0.42, 95% CI: 0.19–0.91; *p* = 0.027).

The pattern of associations differed across age categories. The difference between participants aged 26–35 years and those aged 18–25 years was not significant (aOR = 1.01, 95% CI: 0.43–2.36; *p* = 0.979). In contrast, all 91 participants aged 36–49 years reported at least one IPV behavior, resulting in complete separation. Although Firth penalized regression provided a finite estimate for this category (aOR = 485.80, 95% CI: 24.98–9447.76; *p* < 0.001), the extreme point estimate and very wide confidence interval imply significant estimation instability; thus, the magnitude of this estimate should not be interpreted substantively, and the accompanying *p*-value should not be used to support a precise or stable correlation. Participants aged ≥50 years also had increased adjusted odds compared with those aged 18–25 years (aOR = 6.06, 95% CI: 2.53–14.47; *p* < 0.001).

In the modified model, educational level was associated with reporting any IPV behavior in a non-monotonic pattern of association. Participants with secondary education had higher adjusted odds of reporting any IPV behavior than those with less than secondary education (aOR = 7.02, 95% CI: 3.14–15.69; *p* < 0.001), as did those with a bachelor’s degree (aOR = 3.23, 95% CI: 1.55–6.76; *p* = 0.002). Conversely, those with a master’s degree had lower adjusted odds of IPV (aOR = 0.27, 95% CI: 0.10–0.71; *p* = 0.008).

### 3.7. Sensitivity Analysis

Several estimates in the sensitivity model changed significantly in magnitude or direction, with extremely wide confidence intervals. For example, when occupational status was included, the estimate for the 36–49-year age group dropped from an aOR of 485.80 in the original model to 3.04 (95% CI: 0.04–227.18). Estimates of occupational status were also imprecise, with large confidence intervals. These significant changes in magnitude, direction, and precision suggest that the multivariable model is not very robust when occupational status is included. As a result, the sensitivity model is provided as evidence of model instability rather than a confirmatory analysis, and individual *p*-values from this model should not be interpreted as indicating stable correlations. Thus, the simplified model was kept as the primary model.

## 4. Discussion

The aim of this study was to determine the proportion, patterns, and sociodemographic correlates of reported IPV among adults with physician-diagnosed hypertension in Saudi Arabia. Several findings are noteworthy. First, IPV was commonly reported, with 59.0% of participants reporting at least one of the IPV behaviors assessed. Second, of the IPV domains, controlling behavior was the most frequently reported domain (45.0%), followed by emotional/verbal (28.2%), physical (26.8%), and sexual IPV (13.0%). Third, reported IPV behaviors were often multidomain: 33.8% of the sample reported behaviors in two or more domains. Finally, there were considerable sociodemographic variations, particularly by gender, age, marital status, and educational level.

The present sample also exhibited a comparatively high proportion of younger adults, with 30.6% of participants being under the age of 35 and 13.8% being between the ages of 18 and 25. Hypertension is generally more prevalent in older adults; however, it is also becoming more widely recognized in younger adults, as evidenced by previous studies that have reported a significant prevalence in this age group [14,15]. Nevertheless, these estimates are not directly comparable to the current study, as all participants in our study were recruited using non-probability convenience sampling and had hypertension that was diagnosed by a physician. Therefore, the relatively high proportion of younger participants is likely indicative of the composition and recruitment strategy of this study sample and should not be interpreted as a representation of the age distribution of hypertension in the general Saudi population.

This study found a higher proportion of participants reporting any assessed IPV than previously published estimates in Saudi primary-care populations [8,9,10]. However, these figures are not directly comparable because the present IPV assessment was not anchored to a standardized exposure period, whereas previous studies used differing reference periods, including past-year or lifetime measures. IPV prevalence varies widely across countries. For example, the WHO multi-country survey reported that the lifetime prevalence of physical and/or sexual IPV ranged from 15% to 71% across study sites in Africa, Asia, Latin America, and the Pacific [16]. However, direct comparison with these international estimates is also limited, since prior studies were notably heterogeneous in study populations, IPV definitions, measurement methods, and exposure durations, with some focusing specifically on IPV experienced in the preceding 12 months. By contrast, the present study assessed a wider range of physical, emotional/verbal, sexual, and controlling behaviors and did not restrict reported experiences to a fixed recent timeframe. Accordingly, the 59.0% observed in the current study should be interpreted as the proportion of participants reporting at least one assessed IPV behavior rather than a conventional 12-month or lifetime prevalence estimate. Moreover, our study was limited to individuals with confirmed hypertension, whereas most prior Saudi investigations were conducted among women accessing routine primary-care services. The higher proportion reporting IPV behaviors observed in this study may be attributed to differences in both measurement methods and sample characteristics rather than reflecting a genuinely higher level of IPV reporting in the general Saudi population. Nonetheless, the data indicates a substantial burden of reported IPV behaviors among adults with hypertension and suggests that more attention should be paid to IPV and other psychosocial factors in chronic-disease management.

Controlling behavior was the most frequently reported IPV category in the current study, followed by emotional/verbal, physical, and sexual IPV. This pattern is consistent with that observed in a previous study in which controlling behavior was the most prevalent type of violence among Saudi women, followed by emotional, sexual, and physical abuse [17]. Other Saudi research has also shown the importance of non-physical IPV. For example, Barnawi reported that emotional violence was the most prevalent form of domestic abuse [8], and Abdel-Salam et al. found emotional violence to be more common than physical or sexual violence [9]. IPV domains frequently overlapped in our study, with 33.8% of participants reporting behaviors in two or more domains, including 5.9% who reported all four domains assessed. This is comparable with the Aljouf study, in which 39.3% of women experiencing IPV reported all three types of violence examined [9]. Although differences in IPV definitions, exposure periods, and denominators limit direct numerical comparisons, these findings collectively indicate the multidimensional nature of IPV and the importance of measuring controlling and emotional behaviors alongside physical and sexual violence.

IPV varied by sociodemographic characteristics, although certain associations should be interpreted with caution. In this study, women reported assessed IPV behaviors more frequently than men (75.5% vs. 33.5%). Nevertheless, this observed discrepancy should be interpreted as a distinction in self-reported IPV behaviors, rather than as definitive evidence of a corresponding difference in the underlying occurrence of IPV. This is especially true in specific sociocultural contexts, where the disclosure of sensitive experiences may vary by gender. Furthermore, the instrument was adapted for gender-neutral administration; however, it was derived from a UNECE survey that was originally designed for women and has not undertaken comprehensive psychometric validation specifically for Saudi men and women. Consequently, the identified gender disparity must be regarded with caution. While the study did not investigate whether the context, severity, consequences, or patterns of perpetration varied by gender, IPV behaviors were nevertheless reported by both men and women. Age showed a complex pattern, in part because all participants aged 36–49 years reported at least one assessed IPV behavior, resulting in complete separation and a highly imprecise adjusted estimate. Therefore, the extent of this association should not be interpreted literally. Educational level showed a non-monotonic and inconsistent association with reported IPV behaviors, and the direction of the adjusted estimates varied across educational categories. These findings do not indicate a clear educational gradient and should be interpreted cautiously given the exploratory nature of the multivariable analyses. Previous Saudi studies have likewise reported variable associations between educational attainment and IPV. For example, Barnawi reported an independent association between higher female education and domestic violence [8]. Reviews of Saudi evidence have also found inconsistent relationships between educational attainment and IPV across studies [18,19]. Married participants reported IPV more often than divorced participants, whereas in the Taif study, being divorced was associated with a higher risk of IPV [7]. Therefore, these sociodemographic findings should be interpreted with caution, particularly given differences in study populations and IPV assessment, and the instability of certain adjusted estimates because of limited data and overlap between age and employment status.

This study contributes to the cardiovascular health literature by developing a more detailed characterization of reported IPV behaviors among people diagnosed with hypertension, rather than just analyzing IPV as a binary exposure. In particular, the data suggest high proportions of reported controlling behaviors and multidomain IPV in this clinical population and reveal sociodemographic disparities in IPV reporting. This domain-specific definition points out the need to include psychological and interpersonal variables in descriptions of the larger health environment for patients receiving therapy for hypertension. However, our data are unable to ascertain whether IPV is more prevalent among individuals with hypertension than among those without hypertension or whether IPV contributes to the development of hypertension, as all participants in the present study had hypertension and no normotensive comparison group was recruited.

Any cardiovascular implications stated below should be interpreted contextually, based on previous literature, rather than as effects established by this study. Although the present study was not intended to assess blood pressure control, identification of IPV may be clinically important for patients with hypertension, since successful hypertension management relies on continued drug adherence, frequent monitoring, and self-care engagement. IPV has been related with poor adherence to treatment among those with chronic conditions including hypertension and may impact healthcare utilization and drug use [20]. Previous research has proposed chronic psychosocial stress and its accompanying neuroendocrine and autonomic responses as potential mechanisms linking IPV exposure to blood pressure outcomes [4]. These mechanisms were not addressed in the current investigation and should thus be considered contextual rather than explanatory of the findings presented here.

These findings should be interpreted as implications for clinical awareness and future service development, rather than as specific recommendations for IPV screening or management. First, the proportion reporting IPV behaviors was high, suggesting that psychosocial and interpersonal circumstances should be considered in the care of patients with chronic cardiovascular disease. People with hypertension typically seek primary-care and cardiovascular services, which may offer opportunities for appropriately trained healthcare staff to identify IPV and facilitate access to support. The current study did not investigate the effectiveness of IPV screening, referral pathways, treatment adherence, or blood pressure control, thus no conclusions about these clinical outcomes can be drawn from the current data.

Second, the importance of controlling and emotional/verbal behaviors means that case-finding efforts should not be limited to visible injuries or physical assault. Questions about coercive control, social restriction, financial control, emotional abuse, and sexual coercion may help uncover experiences that might otherwise go unnoticed. This is especially relevant given prior Saudi data indicating low rates of formal help-seeking among people experiencing IPV; in the Taif study, none of the women who experienced IPV reported it to a family physician or a women’s protection agency [7].

At the same time, these findings should not be interpreted as support for indiscriminate screening in the absence of appropriate systems of care. Any clinical approach to IPV should prioritize privacy, confidentiality, patient autonomy, culturally sensitive communication, staff training, and clear pathways to appropriate health, social, and protective services. Rather, the findings indicate that IPV is a potentially important psychosocial problem in this hypertensive clinical population and warrants increased attention within integrated chronic-disease management.

From a scientific standpoint, prospective studies are needed to elucidate the association between IPV and hypertension, including the timing, frequency, duration, and severity of exposure. Recent meta-analytic data indicate an association between lifetime IPV and hypertension, but they also reveal high variability and a predominance of cross-sectional research [6]. Therefore, future Saudi studies should employ standardized IPV instruments and well-defined exposure periods, ideally recruiting representative samples that allow for comparison between individuals with and without hypertension.

This study has several strengths. It included 471 participants with physician-diagnosed hypertension and surpassed the predefined minimum sample size. Unlike most prior Saudi IPV studies, which have focused mainly on women, this study included both women and men and provided information on IPV reporting across genders. Another strength was the assessment of IPV through behaviors in four distinct domains rather than a single overall question, which enabled characterization of individual behaviors, domain-specific reporting, and multidomain exposure. The distinction between single-domain and multidomain reporting was particularly informative, with 33.8% of participants reporting behaviors across two or more IPV domains, demonstrating the additional information provided by this multidimensional approach. Finally, important statistical challenges, including complete separation and multicollinearity, were explicitly assessed and addressed using Firth bias-reduced penalized logistic regression and sensitivity analysis.

Nonetheless, several limitations should also be noted. First, the cross-sectional design precludes assessment of temporal or causal relationships between IPV and hypertension. Thus, the data describes IPV among people with existing hypertension and should not be taken as evidence that IPV causes hypertension. Second, recruitment was based on non-probability convenience sampling through selected healthcare institutions and charitable organizations, which limits generalizability to the wider hypertensive population in Saudi Arabia. Accordingly, the observed 59.0% represents the proportion of participants who reported at least one assessed IPV behavior within this convenience sample and should not be regarded as an estimate of IPV prevalence throughout Saudi Arabia’s adult hypertensive population. In the Saudi sociocultural context, marriage is the most widely recognized form of intimate partnership, while non-marital romantic relationships may be less publicly disclosed due to the prevailing cultural norms [1]. However, IPV can also occur in non-marital intimate relationships. The study may have underrepresented IPV occurring in non-marital relationships or among individuals who had previously experienced IPV in a dating relationship but were single at the time of recruitment due to the exclusion of individuals who were single at that time. As a result, this eligibility criterion may have introduced selection bias and limited the generalizability of the findings to a broader range of intimate partnerships. Third, IPV was self-reported, and it involves highly sensitive experiences; therefore, recall bias, under-reporting, and differential disclosure because of stigma, privacy concerns, or differing perceptions of abusive behaviors are plausible. Furthermore, the IPV behavior categories were not consistently linked to a standardized exposure period, such as the previous 12 months or a formally defined lifetime interval. As a result, the reported 59.0% should be considered as the proportion of individuals who reported at least one evaluated IPV behavior, rather than as an estimate based on a standardized 12-month or lifetime reference period. This prevents direct comparison with studies with clearly defined exposure durations. Requiring responses to all questionnaire items may also have disproportionately discouraged participation by people uncomfortable answering IPV-related questions. Fourth, the study assessed the occurrence of individual behaviors but not their frequency, duration, chronicity, partner context, or within-domain severity. Therefore, the multidomain variable captures the number of IPV domains reported, not the severity of violence. Additionally, although the study included both women and men, it did not examine whether the context, severity, consequences, or patterns of perpetration differed by gender. The IPV assessment also utilized an existing survey module from the UNECE that was translated, back-translated, reviewed by experts, pilot-tested, and evaluated for internal consistency. However, the gender-neutral adapted instrument has not undergone comprehensive psychometric validation for Saudi men and women, and formal cognitive interviewing was not performed. Although the instrument demonstrated good internal consistency in the present sample, the Cronbach’s alpha coefficients should not be interpreted as evidence of construct validity or unidimensionality. Several UNECE items and frequency-related components were modified, merged, or omitted. Because IPV classification was based on endorsement of at least one assessed behavior, these adaptations may have affected which participants were classified as reporting IPV and may limit direct comparability with studies using the full UNECE instrument. Finally, several sociodemographic groups had very sparse or completely separated outcome patterns. In addition, we observed high multicollinearity between age and occupational status, and the sensitivity model became highly unstable with the inclusion of the occupational variable. Accordingly, the multivariable regression results should be interpreted as exploratory, especially for the estimates of age and occupation. These findings suggest that the model’s robustness is limited; thus, the magnitude and precision of the estimates should be prioritized over individual *p*-values, and the sensitivity-model results should not be interpreted as confirmatory evidence of associations. Confirmation in larger and more heterogeneous datasets with sufficient data across sociodemographic strata is required.

## 5. Conclusions

In this Saudi sample, reported IPV behaviors were common among people with physician-diagnosed hypertension, with almost six in ten participants reporting at least one of the IPV behaviors assessed. Because these behaviors were not assessed within a standardized exposure period, this proportion should not be interpreted as a conventional 12-month or lifetime prevalence estimate. Controlling behavior was the most frequently reported domain. About a third of participants reported IPV in two or more domains, indicating its multifaceted nature. There was also considerable variation in IPV reporting across sociodemographic groups, although some adjusted associations were uncertain because of separation and overlap among participant characteristics. These findings provide a descriptive characterization of reported IPV behaviors among adults with hypertension. Because the study did not include a normotensive comparison group and used a cross-sectional design, the findings cannot determine whether IPV is more common among individuals with hypertension or whether IPV contributes to the development of hypertension.

These results add to the Saudi IPV literature in two ways: by describing IPV in a hypertensive population and by examining individual behaviors, domain-specific IPV, and multidomain exposure rather than relying on a single overall estimate of IPV reporting. They support the need for greater recognition of interpersonal and psychosocial factors in chronic-disease care, as well as larger, representative, longitudinal studies using standardized IPV measures to further investigate the relationship between IPV, hypertension, and broader cardiovascular health.

## Figures and Tables

**Table 1:** Sociodemographic characteristics of the participants (*n* = 471).

Characteristic	*n* (%)
**Gender**	
Women	286 (60.7)
Men	185 (39.3)
**Age group, years**	
18–25	65 (13.8)
26–35	79 (16.8)
36–49	91 (19.3)
≥50	236 (50.1)
**Marital status**	
Married	366 (77.7)
Divorced	105 (22.3)
**Educational level**	
Less than secondary	106 (22.5)
Secondary	142 (30.1)
Bachelor’s degree	142 (30.1)
Master’s degree	81 (17.2)
**Occupational status**	
Housewife/unemployed	128 (27.2)
Employed	175 (37.2)
Student	54 (11.5)
Retired	114 (24.2)
**Partner educational level**	
Less than secondary	185 (39.3)
Secondary	178 (37.8)
Bachelor’s degree	27 (5.7)
Master’s degree	81 (17.2)
**Partner occupational status**	
Housewife/unemployed	184 (39.1)
Employed	208 (44.2)
Student	79 (16.8)

**Footnote:** Data are presented as frequency (n) and percentage (%). Percentages may not sum to 100% because of rounding.

**Table 2:** Proportion of participants reporting individual IPV behaviors (*n* = 471).

IPV Domain and Behavior	Yes, *n* (%)
**Physical IPV**	
Slapped or had something thrown that could hurt	112 (23.8)
Pushed, shoved, or had hair pulled	84 (17.8)
Kicked or dragged	53 (11.3)
Choked or burned on purpose	33 (7.0)
**Emotional/verbal IPV**	
Insulted or made to feel bad about oneself	124 (26.3)
Belittled or humiliated in front of others	114 (24.2)
Scared or intimidated on purpose	31 (6.6)
Threatened with relationship instability or separation	61 (13.0)
**Sexual IPV**	
Forced to have sexual intercourse when unwilling	53 (11.3)
Had sexual intercourse out of fear of partner’s reaction	59 (12.5)
Forced to perform an unwanted, offensive, or humiliating sexual act	30 (6.4)
**Controlling behavior**	
Restricted from contact with friends or family	114 (24.2)
Restricted from going outside	111 (23.6)
Prevented from employment or study	112 (23.8)
Controlled or took money without consent	77 (16.3)

**Footnote:** IPV, intimate partner violence. Values represent participants responding “yes” to each behavior. Participants could report more than one behavior and more than one IPV domain; therefore, the categories are not mutually exclusive, and the percentages should not be summed. The denominator for each item was the full sample (*n* = 471).

**Table 3:** Overall, domain-specific, and multidomain patterns of reporting IPV behaviors (*n* = 471).

IPV Measure	*n* (%)	95% CI
**Overall reporting IPV**		
Any reported IPV	278 (59.0)	54.5–63.4
No reported IPV	193 (41.0)	36.6–45.5
**IPV domain**		
Physical IPV	126 (26.8)	23.0–30.9
Emotional/verbal IPV	133 (28.2)	24.4–32.5
Sexual IPV	61 (13.0)	10.2–16.3
Controlling behavior	212 (45.0)	40.6–49.5
**Number of IPV domains reported**		
No IPV domain reported	193 (41.0)	36.6–45.5
One domain reported	119 (25.3)	21.6–29.4
Two domains reported	92 (19.5)	16.2–23.4
Three domains reported	39 (8.3)	6.1–11.1
Four domains reported	28 (5.9)	4.1–8.5
**Multidomain IPV classification**		
No reported IPV	193 (41.0)	36.6–45.5
One IPV domain reported	119 (25.3)	21.6–29.4
≥2 IPV domains reported	159 (33.8)	29.6–38.1

**Footnote:** Any reported IPV was defined as the reporting of at least one of the assessed physical, emotional/verbal, sexual, or controlling behaviors. Domain-specific IPV was defined as the reporting of at least one behavior within the corresponding domain. Domain-specific categories are not mutually exclusive, as a participant may report IPV in multiple domains. Multidomain IPV was defined as the reporting of behaviors across two or more IPV domains. The sum of percentages may not be precisely 100% because of rounding. 95% confidence intervals (CI) were calculated using the Wilson score method. CI, confidence interval.

**Table 4:** Bivariable associations between sociodemographic characteristics and reporting any assessed IPV behavior among study participants (*n* = 471).

Characteristic	No Reported IPV Behavior, *n* (%)	Any Reported IPV Behavior, *n* (%)	χ^2^ (df)	*p*-Value
**Gender**				
Women	70 (24.5)	216 (75.5)	**81.975 (1)**	**<0.001**
Men	123 (66.5)	62 (33.5)		
**Age group (years)**				
18–25	27 (41.5)	38 (58.5)	**100.403 (3)**	**<0.001**
26–35	25 (31.6)	54 (68.4)		
36–49	0 (0.0)	91 (100.0)		
≥50	141 (59.7)	95 (40.3)		
**Marital status**				
Married	130 (35.5)	236 (64.5)	**20.218 (1)**	**<0.001**
Divorced	63 (60.0)	42 (40.0)		
**Educational level**				
Less than secondary	71 (67.0)	35 (33.0)	**69.939 (3)**	**<0.001**
Secondary	25 (17.6)	117 (82.4)		
Bachelor’s degree	52 (36.6)	90 (63.4)		
Master’s degree	45 (55.6)	36 (44.4)		
**Occupational status**				
Housewife/unemployed	25 (19.5)	103 (80.5)	**47.620 (3)**	**<0.001**
Employed	70 (40.0)	105 (60.0)		
Student	27 (50.0)	27 (50.0)		
Retired	71 (62.3)	43 (37.7)		
**Partner’s educational level**				
Less than secondary	72 (38.9)	113 (61.1)	**26.404 (3)**	**<0.001**
Secondary	76 (42.7)	102 (57.3)		
Bachelor’s degree	0 (0.0)	27 (100.0)		
Master’s degree	45 (55.6)	36 (44.4)		
**Partner’s occupational status**				
Housewife/unemployed	96 (52.2)	88 (47.8)	**21.120 (2)**	**<0.001**
Employed	79 (38.0)	129 (62.0)		
Student	18 (22.8)	61 (77.2)		

**Footnote:** df, degrees of freedom. Data are presented as *n* (%), with percentages calculated within each sociodemographic category (row percentages). Bivariable associations were assessed using Pearson’s chi-square test, and assumptions were verified based on expected cell counts. Categories in which 100% of participants reported IPV behaviors represent the observed data, with all participants in those categories reporting at least one IPV behavior A two-sided *p* value < 0.05 was considered statistically significant. Bold values indicate statistically significant associations.

**Table 5:** Crude and adjusted Firth logistic regression analyses of sociodemographic factors associated with any IPV (*n* = 471).

Characteristic	Crude OR (95% CI)	*p*-Value	Primary Model aOR (95% CI)	*p*-Value	Sensitivity Model aOR (95% CI)	*p*-Value
**Gender**						
Women	1.00 (Reference)	—	1.00 (Reference)	—	1.00 (Reference)	—
Men	**0.16 (0.11–0.25)**	**<0.001**	**0.09 (0.04–0.18)**	**<0.001**	**0.003 (0.0001–0.056)**	**<0.001**
**Age group (years)**						
18–25	1.00 (Reference)	—	1.00 (Reference)	—	1.00 (Reference)	—
26–35	1.53 (0.77–3.02)	0.225	1.01 (0.43–2.36)	0.979	**0.008 (0.0002–0.269)**	**0.007**
36–49	**130.71 (7.65–2232.29)**	**<0.001**	**485.80 (24.98–9447.76)**	**<0.001**	3.04 (0.04–227.18)	0.613
≥50	**0.48 (0.28–0.84)**	**0.010**	**6.06 (2.53–14.47)**	**<0.001**	**0.031 (0.001–0.835)**	**0.039**
**Marital status**						
Married	1.00 (Reference)	—	1.00 (Reference)	—	1.00 (Reference)	—
Divorced	**0.37 (0.24–0.58)**	**<0.001**	**0.42 (0.19–0.91)**	**0.027**	0.38 (0.10–1.36)	0.135
**Educational level**						
Less than secondary	1.00 (Reference)	—	1.00 (Reference)	—	1.00 (Reference)	—
Secondary	**9.28 (5.14–16.74)**	**<0.001**	**7.02 (3.14–15.69)**	**<0.001**	**7.82 (2.55–23.99)**	**<0.001**
Bachelor’s degree	**3.47 (2.05–5.89)**	**<0.001**	**3.23 (1.55–6.76)**	**0.002**	**2.41 (1.03–5.64)**	**0.042**
Master’s degree	1.62 (0.89–2.93)	0.115	**0.27 (0.10–0.71)**	**0.008**	**0.011 (0.0005–0.225)**	**0.004**
**Occupational status**						
Housewife/unemployed	1.00 (Reference)	—	—	—	1.00 (Reference)	—
Employed	**0.37 (0.22–0.63)**	**<0.001**	—	—	24.28 (0.92–638.63)	0.056
Student	**0.25 (0.12–0.49)**	**<0.001**	—	—	**0.005 (0.0001–0.205)**	**0.005**
Retired	**0.15 (0.08–0.27)**	**<0.001**	—	—	**54.21 (2.29–1280.63)**	**0.013**

**Footnote:** OR, odds ratio; aOR, adjusted odds ratio; CI, confidence interval. The dependent variable was any IPV (0 = no IPV; 1 = any IPV). Crude estimates were obtained from separate univariable Firth logistic regression models. The primary model simultaneously adjusted for gender, age group, marital status, and educational level. The sensitivity model additionally included occupational status. Reference categories were women, age 18–25 years, married status, less than secondary education, and housewife/unemployed occupational status. Wald 95% CIs and two-sided Wald *p* values are presented. Small ORs are reported to three significant figures to avoid rounding to zero. Bold values indicate *p* < 0.05.

## Data Availability

Anonymized data are available upon a reasonable request and subsequent approval by the ethics committee.
